# Astrocytopathy in Wernicke Encephalopathy and Neuromyelitis Optica Spectrum Disorder. Pathogenic Differences With Occasional Clinical and Neuroimaging Overlap. A Review

**DOI:** 10.1007/s11910-026-01497-z

**Published:** 2026-04-25

**Authors:** Xiaojun Zhang, Jorge C Kattah

**Affiliations:** 1 Department Of Neurology, Wexner Medical Center, The Ohio State University, Columbus, USA; 2 Department of Ophthalmology and Visual Sciences, Wexner Medical Center, The Ohio State University, Columbus, USA; 3 Department Of Neurology, University of Illinois College of Medicine, Peoria, USA

**Keywords:** Astrocytopathy, Wernicke encephalopathy (WE), Thiamine deficiency, Neuromyelitis optica spectrum disorder (NMOSD), Optic neuropathy, Overlap between WE and NMOSD

## Abstract

**Purpose of the Review:**

There is an increasing number of single case reports describing patients with findings compatible with thiamine deficiency, in whom rapid replacement of thiamine fails to induce improvement. Further work-up identifies positive aquaporin-4 antibodies (AQP-4) leading to a final diagnosis of neuromyelitis optica spectrum disorder (NMOSD). Occasionally, NMOSD is the initial diagnosis, but it turns out that the patient has thiamine deficiency. Similarly, these two diagnoses may overlap, particularly with area postrema lesions and in other cases of protracted vomiting.

**Recent Findings:**

We reviewed the literature to attempt further clarification for this clinical overlap. The common denominator is the astrocyte, as cytotoxic edema due to impaired mitochondrial dysfunction and lactic acidosis in instances of thiamine deficiency causes downregulation of the AQP-4 receptor leading to vasogenic edema and breakdown of the blood-brain-barrier (BBB). Similar dysfunction of the AQP-4 receptor occurs because of IgG binding antibodies in NMOSD. Impaired glutamate transport in the astrocytic podocytes regardless of the AQP4 receptor etiologic mechanism causes excitotoxicity.

**Summary:**

Awareness of this clinical overlap is critical to initiate timely treatment in thiamine deficiency states and NMOSD

## Introduction

Wernicke encephalopathy (WE) and Neuromyelitis Optica Spectrum Disorder (NMOSD) were described in the late 19th century, by Carl Wernicke in 1881 [1, 2], and by Eugẻne Devic in 1894, respectively [3] Their clinical characteristics in the majority of cases are substantially different, NMOSD often affects the optic nerves and spinal cord, [3] WE only rarely affects the optic nerves, [4, 5] and exceptionally the spinal cord [6, 7]. However, when NMOSD affects the brainstem, diencephalon and cerebrum, the localization of lesions coincides with what is often observed in WE. This similarity results from early compromise of astrocytes and secondary breakdown of the blood brain barrier (BBB). Consequently, there is potential overlap in clinical findings despite different etiologies and pathogenesis.

The initial clinical/ anatomic -pathological observations in WE demonstrated selective hemorrhagic lesions in the upper brainstem/diencephalon affecting primarily the glia and myelin, and to a lesser extent neurons, thus the name “*polioencephalitis hemorrhagica superioris*”[1, 2]. A clinical triad was recognized in the original three patients: encephalopathy, ataxia and ophthalmoplegia, and presumed an inflammatory etiology. Korsakoff independently recognized another aspect of this disorder associated with alcohol dependence [8]. Subsequently, in the ensuing 150-years identified biochemical, morphological and more recently neuroimaging findings, established the molecular structure [9] and metabolic role of thiamine, and elucidated selective neuronal vulnerability responsible for the changes observed in pathological [1, 10, 11] and imaging studies [12]. In addition, it highlighted the possibility for early effective therapeutic intervention. Since the original description, NMOSD has been classified as a demyelinating disorder involving antibodies against the aquaporin 4 receptor (AQP-4) in astrocytes [13]. The purpose of our review is to explore the evidence for an initial astrocytopathy as an instrumental contributor to the pathogenesis and initial lesion selectivity in instances of thiamine deficiency with WE and NMOSD. Despite essential differences between NMOSD with an autoimmune/demyelinating etiology versus the primary biochemical and excitotoxic etiology in thiamine deficiency, knowledge of the AQP-4 receptor dysfunction in astrocytes in NMOSD, contributes to the understanding of the initial metabolic thiamine-deficiency associated BBB disruption, and the few reports of diagnostic overlap [14–19].

**Fig. 1:**
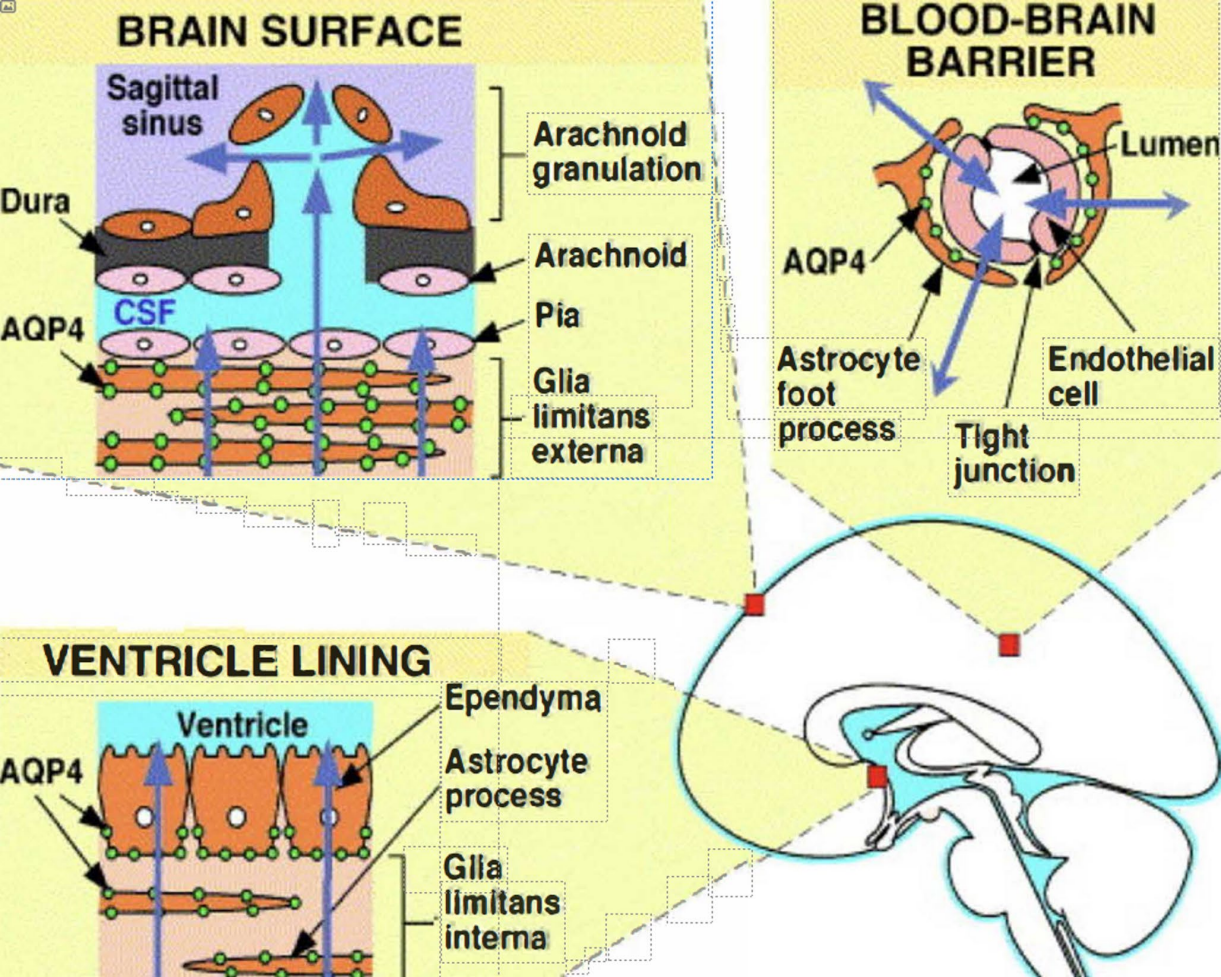
AQUAPORIN 4 RECEPTOR LOCALIZATION IN THE BRAIN SURFACE, VENTRICULARLINNING AND BLOOD BRAIN BARRIER. Adapted from Verkman, et al, 2006. Biochim Biophys Acta 2006; 1758:1085–1093, with permission from Elsevier

To enhance awareness between the clinical and neuroimaging WTE/NMOSD overlap, we aim to increase understanding regarding shared neuroanatomical locations, altered BBB function and common glia susceptibility [20]. Even though he pathogenesis is different, a review of recent basic science progress potentially shades light on a common initial injury targets (Fig. 1) [21]. For clarity, we divide the discussion into brief sections:

## Astrocyte Dysfunction in WE and NMOSD

The role of thiamine in brain metabolism is well defined and represents the basis for the consistent lesion localization that causes the typical of WE clinical manifestations. Because thiamine deficiency targets primarily astrocytes, it is associated with both cytotoxic and vasogenic edema, as well as disruption of the BBB [22–24]. Cytotoxic edema results from combined metabolic derangement and excitotoxicity, leading to irreversible neuronal death if untreated. These lesions have been demonstrated consistently by neuroimaging in about 50% of patients [12, 25] In addition, the BBB changes share common CNS lesion localization with classic autoimmune astrocytopathy (NMOSD) with preferential periventricular localization. Even though NMOSD involves extensive inflammation and demyelination, which is less conspicuous in WE, their common lesion localization prompted us to review studies on the role of thiamine and the AQP-4 receptor [22, 26] In astrocyte cell cultures, thiamine deficiency causes downregulation of the AQP-4 receptor leading to edema, and these changes are reversible with replenishment of thiamine [20] Hypothetically, cytotoxic edema will result in early BBB changes, and if not corrected, eventually results in irreversible neuronal death.

We now know that the classic WE triad is infrequent [27] and found after prolonged thiamine deficiency, emphasizing the need for early diagnosis, and ideally before encephalopathy develops. We became aware of the fact that early manifestation of thiamine deficiency is subradiographic. Interestingly, these cases are rapidly responsive to treatment and have excellent prognosis [28, 29].

## Optic Nerve Lesions. Clinical Feature Overlaps Between NMOSD and WE

Acute bilateral Optic Neuropathy: Acute severe bilateral optic neuropathy with longitudinal extensive optic nerve lesions commonly affecting the optic chiasm is one of the core clinical features of NMOSD [30, 31]. Optic neuropathy in WE is distinctly uncommon, though present in two of the three original patients by reported by Wernicke [2]. In stark contrast to NMOSD, optic neuropathy in WE has been reported only in a few patients by De Wardener among WWII prisoners of war, [32] and not found among 245 cases studied in the largest 1978 series of WE ever reported by Victor, Adams and Collins [1]. Li et al reviewed the literature and found 13 instances of optic neuropathy and vision loss in cases of WE [4]. Nachbor and colleagues, in 2023 reported a patient with WE and bilateral optic neuropathies as the initial presentation. PubMed-review of the literature included only nine cases of WE presenting with acute optic neuropathy in the preceding 20 years [5]. All cases were bilateral. Most cases occurred in women (70%) with an average age of 37 years. The average time from vision loss to presentation was 17 days (range, 3–63 days), with 80% presenting within the first 2 weeks. Most cases presented with optic disc edema (70%), and nystagmus [4]. Of note, out of these 9 cases, only 2 of them had history of alcohol dependence. The etiology of thiamine deficiency in the remaining cases were gastrointestinal conditions related to multiple medical comorbidities such as gastric bypass, hyperemesis gravidarum, chronic diarrhea and side effects of chemotherapy. Different from AQP-4 , in cases of NMOSD, visual function outcome of optic neuropathy due to vitamin B1 deficiency is usually good once patients receive high-doses of vitamin B1 supplementation [4, 5, 31] Bilateral optic disc edema is present in 20 to 31 % of NMOSD patients and is found in 70% of reported cases of WE. This suggests that the prelaminar, unmyelinated optic nerve is a susceptible structure in cases of thiamine deficiency. 

Discrete peripapillary hemorrhages, and severe bilateral loss of vision are common in WE [4]. For example, patient 11, Table 1 provides a characteristic example of an acute bilateral optic neuropathy. Here, ocular computed tomography (OCT) showed increased bilateral retinal nerve fiber layer (RNFL) thickening. In contrast to the classic NMOSD, MRI showed normal-sized retrobulbar optic nerves, with increased signal in axial/coronal T2 sequences, but without post-contrast enhancement. This finding is in stark contrast with optic neuritis in NMOSD where it is common to find longitudinally extensive retrobulbar optic nerve enhancement and frequent involvement of the optic chiasm. Confirmatory pre-treatment decreased whole blood thiamine-diphosphate predicted rapid, albeit partial improvement with high-dose thiamine supplementation. In contrast, there was slow nystagmus and ataxia improvement over weeks (Table 1). One critical difference between WE and NMOSD was the lack of the anticipated NMOSD retrobulbar optic nerve enhancement, days later, the AQP4-receptor antibody resulted negative.

The mechanisms underlying severe optic neuropathy with optic disc edema in WE are not completely understood. To our knowledge there is no histologic study performed outside the original description by Wernicke [2]. However, thiamine deficiency is the most common nutritional optic neuropathy associated with optic disc edema. Presumably, CT results from compromise of optic nerve astrocytes with downregulated AQP-4 receptor causing disc edema [33] leading to axonal swelling, inflammation and secondary ganglion cell loss. In addition, in an extensive review of the ocular manifestations of WE, mitochondrial dysfunction was proposed as a probable mechanism because thiamine is a key factor in cellular energy metabolism [31, 34] Other nutritional optic neuropathies preferentially target optic nerve axons, leading to ganglion cells loss. In addition, to the retrolaminar demyelination process. OCT is widely used as a biomarker in neurodegenerative and inflammatory central nerve system disorders and optic neuropathy with different causes. Reports of WE optic neuropathy with OCT findings are limited, often due to patients inability to cooperate [5] In patient 11, Table 1, the RNFL thickening lessened on a second study after treatment, with residual bilateral and substantial generalized ganglion cell /internal limiting layers (GCL/IPL) thinning.

**Table 1:** Neuromyelitis optica spectrum disorder mimicking thiamine deficiency

Report	Age	Clinical findings	MRI	Response to thiamine	Final diagnosis
1 Shan F, et al. 2016. Case A Studied in 2010 *doi.org/10.3109/00207454.2015.1084619*	20	Somnolence, weakness in both lower extremities	Periventricular Diencephalic Increased signal No contrast enhancement	Treated with thiamine. Improved when treated for NMOSD	AQP-4 + NMOSD Normal CSF
2. Shan F, et al. 2016. Case B Studied in 2011 *doi.org/10.3109/00207454.2015.1084619*	62	History of protracted vomiting and vertigo for 2 weeks. Then somnolence and weakness	Periventricular Diencephalic Increased signal No contrast enhancement	Treated with thiamine. Improved when treated for NMOSD	AQP-4 + NMOSD. Normal CSF
3. Shan F, et al. Case C. Studied in 2014 *doi.org/10.3109/00207454.2015.1084619*	48	Somnolence, Hallucinations, Intractable hiccups. Chronic alcohol dependence Known AQP4	Periventricular Diencephalic Increased Signal. No contrast Periventricular Diencephalic Increased signal enhancement	Treated with thiamine. Improved when treated for NMOSD	AQP-4 + NMOSD. No spinal cord lesions
4.Shan F, et al. Case D. Studied in 2014 *doi.org/10.3109/00207454.2015.1084619*	26	Somnolence, hallucinations, intractable hiccups. Chronic alcohol dependence Known AQP4	Periventricular Diencephalic and floor of the fourth ventricle Increased signal No contrast enhancement	Treated with thiamine. Improved when treated for NMOSD	AQP-4 + NMOSD. No spinal cord lesions
5. Zhang D, et al. 2019. Neurology \India DOI 10.4103//0028-3886.273629	28	Poor nutrition and vomiting. Diplopia Unsteadiness Left abducens palsy and horizontal gaze evoked nystagmus	Periventricular Diencephalic, and periaqueductal gray Increased signal No contrast enhancement	Improvement initially. Six months later myelopathy and multiple spinal cord lesions AQP-4 positive	AQP-4 + NMOSD
6. Karri M, et al. 2020 J R Coll Physicians Edinb 2020; 50: 305 DOI 10.4997/JRCPE.2020. 320	23	Episodic vomiting, treated with intravenous thiamine and improved for 1 week; then worsened. History of unilateral optic neuritis 4 months earlier	Increased periventricular Signal Diencephalon Periaqueductal gray Midbrain Vestibular nuclei and area postrema. No contrast enhancement	Treated with thiamine with transient improvement Then improved when treated for NMOSD	AQP-4 + NMOSD Normal CSF. Had previous optic neuritis
7. Shirah BH, et al. 2023. MSARD. 2022. 10,436 DOI: 10.1177/19418744241228004	30	Vertigo and vomiting for 1 month. Diplopia, ptosis, ataxia	Increased MRI signal in periventricular diencephalon, and periaqueductal grey	Treated with thiamine Improved when treated for NMOSD	AQP-4 + NMOSD
8. Supahiah P, et al. 2023; 43 e293-295 *DOI*: 10.1097/WNO.0000000000001444	20	Diplopia Blurred vision	Increased signal optic nerve, hypothalamic periventricular brainstem, spinal cord	Intravenous thiamine without improvement	AQP 4 + NMOSD
9. Lynch S, et al. The Neurohospitalist 2024; 14: 213-217 doi:*10.1177/19,418,744,241,228,004*	39	Increased Somnolence, fatigue and forgetfulness, bilateral abducens palsy, and global duction restriction	Increased signal in MRI involving periventricular diencephalon, periaqueductal gray, mammillary bodies, fourth ventricle	Pre-treatment serum thiamine was normal. Improved when treated NMOSD	AQP-4+ NMOSD
10. Sato T, et al. 2024. Cureus DOI: 10.7759/cureus 0.63920	29	Headache, nausea, loss of appetite. Diplopia. Somnolence. Right third nerve palsy	Increased signal in MRI in periventricular Diencephalon Periaqueductal and area postrema	Pre-treatment serum thiamine was normal. CSF pleocytosis (26 WBC’s) Improved when treated for NMOSD	AQP-4+ NMOSD Normal serum thiamine level
11. Zhang Y, et al. 2026. Novel Library *	18	Protracted Vomiting Bulimia. Unable to stand, Somnolence. Severe bilateral visual loss. Bilateral optic disc edema with few scattered retinal hemorrhages. Upbeat nystagmus	Increased signal in both optic nerves. No enhancement. Increased signal in the area postrema with contrast enhancement. OCT with RNFL thickening	Low pre-treatment serum thiamine. Patient Improved with thiamine and caloric replacement. OCT with RNFL thinning. Slow Nystagmus Improvement	AQP-4: Negative Low serum thiamine level

## Astrocytes in the Optic Nerve

Astrocytes are present in both unmyelinated and myelinated optic nerve axons. Importantly, astrocytes directly unsheathe these optic nerve axons. This is because the surface and sieve-like pores of the collagenous plates of the primate lamina cribrosa are lined with astrocytes, which separate the connective tissue septa within the lamina cribrosa from the nerve axon bundles [35] Theoretically, in WE - optic neuropathy these astrocytes are primarily affected. Cultures of vitamin B1 deprived rat astrocytes respond to thiamine deficiency - induced swelling by downregulating AQP-4 levels, [33] a factor that is not present in other nutritional neuropathies. Additional factors in WE involve mitochondrial dysfunction which increases the severity of the visual loss. Delayed treatment has been associated with only partial recovery of visual function. Reasons for the infrequent optic nerve lesion in WE are not clear.

## Area Postrema Syndrome (APS) and Other Related Brainstem Lesions in NMOSD and WE

Conspicuous area postrema involvement with overt MRI finding can be observed in both NMOSD and WE. The dorsal medulla oblongata is frequently affected in NMOSD, resulting in the area postrema syndrome which is characterized by intractable acute or subacute nausea, vomiting and/or hiccups (either in combination or isolated; constant or episodic). The area postrema syndrome is one of the core NMOSD diagnostic criteria, [30] and more frequently found in instances of AQP4-antibody positive NMOSD [30]. Prolonged vomiting in cases of NMOSD with involvement of the area postrema may also be complicated by simultaneous thiamine deficiency [36]. In the patient with WE and bilateral optic neuropathy, local BBB in the dorsal medulla involved the nucleus intercalatus of Staderini, near the area postrema. (Patient 11, Table 1).

The area postrema is one of the circumventricular organs, which renders it as a common NMOSD target. The nucleus intercalatus lays near, and upbeat nystagmus (UBN) is a frequent and possibly underreported finding in medullary lesions in NMOSD [37]. In addition,, the dorsal midbrain, the tectal plate and the periaqueductal gray are commonly affected, ^12, 25^ In WE, lesions in these locations are frequent, and UBN has been documented in WE series [38] UBN often improves, particularly when co-existent with horizontal nystagmus [29]. However, UBN often makes a transition to downbeat nystagmus (DBN) and may remain unchanged for years [38].

In addition, symptomatic narcolepsy, and acute diencephalic clinical syndrome are less common clinical presentations in NMOSD. Unlike NMOSD, spinal cord involvement in WE though previously described is rare. Although infrequent, the WE triad of altered mental status, ophthalmoplegia, and ataxia may be present in NMOSD as well (Table 1). In addition, both diagnoses may coincide in the same patient [14].

The mechanism for UBN converting to DBN hypothetically involves simultaneous compromise of the nucleus of Staderini and paramedian tract neurons. Whereas the former recovers, the latter does not, as a result, the cerebellar flocculus fails to inhibit the superior vestibular nucleus (SVN) causing an upward ocular drift with a corrective downward fast phase; the SVN should be functioning normally during the process [39]. Conversely, mild non-triggered UBN fluctuations and chronic DBN cases with transient positional triggered UBN [38] are also rarely observed in WE [40]. We did not find reports of UBN converting to DBN in patients with NMOSD, probably because of effective treatment.

## Overlapping Brain MRI Features in WE and NMOSD

While NMOSD has long been considered a disease without brain involvement, current studies have shown that brain MRI abnormalities exist in a notable proportion (50–85%) with characteristic features [41]. These abnormalities affect sites with high AQP-4 receptor expression adjacent to the ventricular system at any level, such as the hypothalamus, and peri ependymal areas surrounding the third and lateral ventricles, cerebral aqueduct, corpus callosum, and dorsal brainstem adjacent to the fourth ventricle. Many typical brain MRI findings correlate with specific clinical findings, such as intractable vomiting and hiccup (linear dorsal medullary lesions) involving the area postrema and nucleus tractus solitarius (NTS). Curiously, mammillary body enhancement, in the post-contrast MRI considered a neuroimaging biomarker of WE, may be present in NMOSD as well [42].

Similarly, MRI findings in cases of WE typically show symmetric T1 hyperintense and T2/FLAIR hyperintense signals in the medial thalamus, mammillary bodies, periaqueductal region, and cerebellum bilaterally [12, 25, 43] Zuccoli and collaborators in 2007 reported MRI findings among 26 WTD patients (Table 2, left column), [12] and later Hiraga and colleagues [44] studying 12 consecutive WE with low vitamin B1 levels found increased signal in several diencephalic and brainstem locations (Table 2 right column). From these data, WE lesion limited to the medulla are rather infrequent. Of interest, besides optic disc edema and retinal hemorrhage - related severe vision loss, central visual pathway involvement with lesions in the optic chiasm, lateral geniculate body, or occipital cortex, have been occasional WE MRI findings [45]. The robust contrast enhancement in NMOSD longitudinal extensive optic neuritis, [46] is not present in WE, though, on occasion post-contrast lesion enhancement is found as well (Table 1).

**Table 2:** MRI findings in wernicke’s encephalopathy

Zuccoli *n*=26 patients	Hiraya *n*=12 Patients
Periventricular 85%	Mammillary /Medial Thalamus 50%
Third Ventricle 85%	Dorsal Midbrain 41.7%
Periaqueductal 65%	Fornix 3 Patients
Mammillary Bodies 58%	Splenium of Corpus Callosum 50%
Tectal Plate 38%	Cortex 2, Vermis 2
Dorsal Medulla 8%	Medulla 1 patient

## Astrocytes: A Potential Shared Lesion Target and Mechanism of CNS Injury in NMO and WE

From previous work in NMOSD and WE, there is shared astrocyte dysfunction and BBB alterations causing periventricular lesion localization [20, 31, 33, 34]. From the neuroimaging perspective, the lesion site selectivity in both NMO and WE [46], clusters in periaqueductal localization in the brainstem and diencephalon. This selectivity might be explained by involvement of astrocytes in regions where the BBB normally is structurally thin (composed of permeable endothelial cells, designed to provide neuroendocrine function) [47]. Local BBB breakdown detected by MRI brain with contrast enhancement in both NMO and WE supports this hypothesis. Table 2 is a summary of most common MRI lesion localization.

## Mechanism of Neuronal Injury Following Alterations of the BBB in WE and NMOSD

Besides astrocytopathy, the pathophysiology of the glial/myelin and neuronal injury in WE involves several factors including primarily impaired function of the Krebs cycle (tricarboxylic acid), with decreased ATP production/utilization in neurons with oxidative stress, lactic acidosis and mitochondrial dysfunction [34]. Decreased level of gamma-aminobutyric acid (GABA) levels, leads to one of the most important mechanisms of injury “e*xcitotoxicity.”* Astrocytes are involved in the transport of glutamate, acting as buffers (The transporters are EAAT1 and EAAT2), found low in experimental rat thiamine deficiency in the thalami, [23, 48] and similarly decreased in human WTD [23] Additional astrocyte important roles includes regulation of K^+^ ions, trafficking of metabolites and brain water homeostasis. On the other hand, NMOSD pathogenesis, involves the binding of AQP4-IgG autoantibodies to water channel (AQP4 receptor) on astrocytes, results in complement- and cell-mediated astrocyte injury, inflammation, demyelination, and neuron loss [13]. Excitotoxicity is undoubtedly a substantial cofactor which amplifies the immune-mediated injury.

The neuropathology of WE show (1) Prominent blood vessels due to proliferation of endothelium and adventitia, (2) Glial proliferation (3) Slight to moderate neuron damage (4) Absent inflammatory cells (5) Rare hemorrhages (6) Restriction of the lesions to the gray matter (7) Remarkably symmetric lesions. There is necrosis without cavitation [1, 11].

## Clinical Overlap Between NMOSD and WE

There are few reports of initial NMOSD misdiagnosis, initially believed to represent WE (Table 1, cases 1–10). Lack of improvement with thiamine replacement pointed to the correct diagnosis, note in Table 1, case 11 featuring an encephalopathic patient who had an initial diagnosis of NMOSD, as a mimic of WE. This patient had two core NMOSD diagnostic criteria with bilateral optic neuropathy and UBN due to area postrema compromise [30]. Finally, there is at least one report of both NMOSD and WE in the same patient [36]. This is not at all unexpected as lesions of the area postrema can clearly protracted vomiting and thiamine deficiency.

## Conclusions

Severe bilateral optic neuropathy is a common presentation of NMOSD, particularly in young patients, but distinctly uncommon in WE. The normal WE retro-orbital optic nerves in MRI of the orbits represents lack of inflammation and points to lesion localization within the prelaminar, non-myelinated optic nerve head in thiamine deficiency [4, 5, 48–50]. The brainstem and diencephalic lesions in both disorders do share BBB changes with contrast enhancement in NMOSD, and occasional in WE with possible diagnostic confusion [48]. This review provides basis to the concept of an initial non-inflammatory astrocytopathy that causes first downregulation of the AQP-4 receptor, excitotoxicity and vasogenic edema, with subsequent compensatory upregulation of the AQP-4 receptor after treatment leading to resolution of the edema in WE. The inflammatory /demyelination in NMOSD, at the peak of the cytotoxic edema phase causes decreased Glutamate transporter function, resulting in added excitotoxicity and neuronal injury; these shared mechanisms play a key role in the pathophysiological process of both WE and NMOSD. Mitochondrial dysfunction with lactic acidosis is a specific cofactor in thiamine deficiency. Finally, spinal cord lesions are frequent in NMOSD and rare in WE [6]. The possibility of therapeutic intervention to control glutamatergic toxicity to our knowledge, [49] has not been investigated to date. 

## Key References

- Lennon VA, Kryzer TJ, Pittock SJ, Verkman AS, Hinson SR. IgG marker of optic-spinal multiple sclerosis binds to the aquaporin-4 water channel. J Exp Med 2005; 202:473–477. 10.1084/jem.20050304.
  - ○ This study identified antibodies against the Aquaporin-4 water channel in NMOSD, exactly 13 years after the discovery of this channel by Peter Agre et al in 1992, which led to the understanding of water transport across membranes, and earned the 2003 Nobel Prize od Chemistry.
- Verkman AS, Binder DK, Bloch O, Auguste K, Papadopoulos MC. Three distinct roles of aquaporin-4 in brain function revealed by knockout mice. Biochim Biophys Acta 2006;1758: 1085–1093. 10.1016/j.bbamem.2006.02.018.
  - ○ This paper illustrates different roles of the Aquporin-4 receptor in cerebral water balance astrocyte migration and neural signal transduction.
- Hazell AS, Sheedy D, Oanea R, et al. Loss of astrocytic glutamate transporters in Wernicke encephalopathy. Glia 2010;58:148–156 10.1002/glia.20908.
  - ○ These authors identified for the first time, loss of astrocytic glutamate receptors in the frontal cortex of patients with Wernicke’s Encephalopathy.
- da Silva APB, Souza DG, Souza DO, Machado DC, Sato DK. Role of Glutamatergic Excitotoxicity in Neuromyelitis Optica Spectrum Disorders. Front Cell Neurosci 2019;13:142. 10.3389/fncel.2019.00142.
  - ○ These authors propose utilization of medications to reduce glutaminergic excitotoxicity in NMOSD, which in principle could improve clinical outcomes in thiamine deficiency as well.

## Data Availability

No datasets were generated or analysed during the current study.
